# Clonal Expansion of T Cells in Abdominal Aortic Aneurysm: A Role for Doxycycline as Drug of Choice?

**DOI:** 10.3390/ijms160511178

**Published:** 2015-05-18

**Authors:** Albert M. Kroon, Jan-Willem Taanman

**Affiliations:** Department of Clinical Neurosciences, Institute of Neurology, University College London, London NW3 2PF, UK; E-Mail: amk@keab.nl

**Keywords:** abdominal aortic aneurysm (AAA), clonal expansion, doxycycline, drug target, matrix metalloproteinases, mitochondrial protein synthesis, T cells

## Abstract

Most reported studies with animal models of abdominal aortic aneurysm (AAA) and several studies with patients have suggested that doxycycline favourably modifies AAA; however, a recent large long-term clinical trial found that doxycycline did not limit aneurysm growth. Thus, there is currently no convincing evidence that doxycycline reduces AAA expansion. Here, we critically review the available experimental and clinical information about the effects of doxycycline when used as a pharmacological treatment for AAA. The view that AAA can be considered an autoimmune disease and the observation that AAA tissue shows clonal expansion of T cells is placed in the light of the well-known inhibition of mitochondrial protein synthesis by doxycycline. In T cell leukaemia animal models, this inhibitory effect of the antibiotic has been shown to impede T cell proliferation, resulting in complete tumour eradication. We suggest that the available evidence of doxycycline action on AAA is erroneously ascribed to its inhibition of matrix metalloproteinases (MMPs) by competitive binding of the zinc ion co-factor. Although competitive binding may explain the inhibition of proteolytic activity, it does not explain the observed decreases of MMP mRNA levels. We propose that the observed effects of doxycycline are secondary to inhibition of mitochondrial protein synthesis. Provided that serum doxycycline levels are kept at adequate levels, the inhibition will result in a proliferation arrest, especially of clonally expanding T cells. This, in turn, leads to the decrease of proinflammatory cytokines that are normally generated by these cells. The drastic change in cell type composition may explain the changes in MMP mRNA and protein levels in the tissue samples.

## 1. Introduction

Abdominal aortic aneurysm (AAA) is characterised by a progressive localised weakening and dilatation of the abdominal aorta. AAA is a common and serious medical condition in elderly people, especially among men [1]. The 2013/2014 results of the National Health Service AAA Screening Programme suggested a 1.25% prevalence of AAA with a diameter larger than 3.0 cm in 65-year-old men who had not had previous AAA surgery [2]. There is a high propensity to rupture when the aneurysm diameter reaches 5.0 to 5.5 cm. The mortality associated with AAA rupture is about 70% [2,3]; the majority of patients will die before they reach the operating theatre due to massive internal bleeding. Small aneurysms are, however, not void of the risk of rupture either [4].

Surgical repair is currently the only available treatment option for AAA. There are two scenarios for surgical intervention: traditional open repair and endovascular repair [5,6,7]. Small, asymptomatic AAAs are normally managed conservatively by regular monitoring. Current guidelines recommend surgical repair when the aneurysm diameter is expanding by more than 1 cm per year or is larger than 5.5 cm. Nevertheless, conservative management may remain the preferred choice for patients at high risk of mortality during surgical treatment.

## 2. Pharmacological Interventions for AAA

A pharmacological treatment that arrests or slows the progression of AAAs, thus reducing the pressure for surgical intervention, would have major advantages for patients as well as the healthcare system. Various drug targets have been considered and numerous attempts to affect these targets have been reported. An overview of these studies is given in Table 1. The advances in mechanistic studies that provide insights into these potential pharmacological treatments of AAA have been reviewed recently [8,9,10]. The authors conclude that, to date, there is no proven pharmacological intervention available for preventing the expansion and rupture of AAA. The observation reported recently that aneurysmal lesions contain clonally expanded T cells [11] prompted us to compare the available evidence for the reported inhibitory effects of doxycycline on AAA with our own experiences in tumour systems [12,13].

## 3. Doxycycline and AAA

In its initial stage, AAA is an inflammatory condition. Roles for B and T lymphocytes have long been recognised in AAA disease [14,15,16]. This has led to the notion that AAA may be a specific antigen-driven T cell disease [17,18] and attempts to characterise the antigens have been published [19]. In a recent review, treatment with the tetracycline antibiotic doxycycline was advocated as a non-invasive therapy for AAA, based on its inhibitory effects on matrix metalloproteinases (MMPs) [10]. A moderate change of MMP activity was confirmed in a clinical trial by Lindeman *et al.* [20] but their main observation concerned a significant reduction of the aortic wall neutrophil and cytotoxic T cell content. The recent finding by Lu *et al.* [11] that aneurysmal lesions contain clonally expanded T cells may indicate that Lindeman *et al.* [20] have actually observed the inhibitory effect of doxycycline on this expansion.

**Table 1:** Targets and pharmacological approaches for a conservative treatment of abdominal aortic aneurysm (AAA).

Targets	Pharmacological Approaches	References
Neutrophils and other blood components	Anti-neutrophil antibodies, TNF-α inhibition, mycophenolate	[21–28] this review
β-Adrenergic blockade	Propranolol	[29–33]
Renin-angiotensin system	TGF-β1, cyclosporine A	[22,24,28,34–47]
Cholesterol metabolism	Various statins and proteases	[43,47–57]
Phospholipid metabolism	5-LO inhibitors, cyclooxygenase-2 inhibitors, EP4 inhibitors	[26,28,58–64]
Peroxisome proliferation-associated receptor (PPAR)	Glitazones	[65,66]
Various proteases: cysteine proteases, serine proteases, matrix metalloproteinases (MMPs), elastase and others	Calpain inhibitors, chymase inhibitors, doxycycline, azithromycin, rhoxithromycin	[24,25,33,39,41,67–70] this review

## 4. Doxycycline and Inhibition of T Cell Clonal Expansion

**Figure 1:**
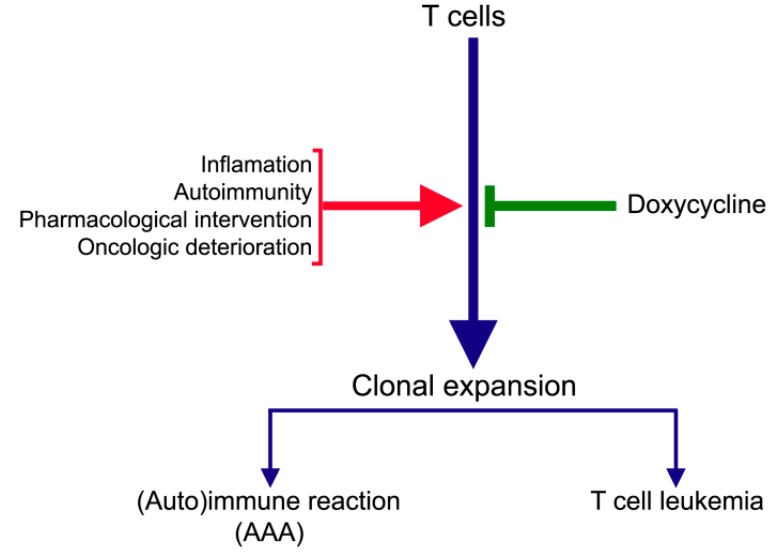
Schematic presentation of the possible causes of clonal expansion of T cells and the inhibition of doxycycline.

Mitochondria, which are descendants of α-proteobacteria, are sensitive to antibacterial agents interfering with ribosomal function, such as doxycycline [71]. Our extensive *in vitro* and *in vivo* investigations on the cytostatic and cytocidic effects of doxycycline and some other tetracycline antibiotics, led us to the idea that inhibition of mitochondrial protein synthesis and the subsequent decrease of the mitochondrial energy-generating capacity is a key target to fight cancer [13]. Tumour growth can be defined as a form of clonal expansion. In a rat T cell leukaemia model, arrest of T cell proliferation through inhibition of mitochondrial protein synthesis by doxycycline has been demonstrated to lead to a total elimination of the tumour [72,73]. It is tempting to speculate that inhibition of mitochondrial protein synthesis is the primary effect of doxycycline in the experimental and clinical studies of AAA. The proposed mechanism is illustrated in Figure 1.

## 5. Doxycycline and Inhibition of Matrix Metalloproteinases (MMPs)

MMPs play a key role in the degradation of extracellular matrix in normal physiological processes, such as embryonic development and tissue remodelling, as well as in pathogenic mechanisms of, e.g., tumour metastasis, arthritis and the formation of AAA [74]. Most members of the MMP family are soluble enzymes, secreted as inactive proproteins, which are activated upon cleavage by extracellular proteinases. A subset of MMPs however, is anchored to the plasma membrane. One of the most widely studied membrane-type MMPs, MT1-MMP, participates in the turnover of various extracellular matrix components and the activation of secreted MMPs [75]. MT1-MMP was reported to have an important function in the macrophage-dependent destruction of the elastin fibre network during AAA formation [76]. The secreted MMP-2 (gelatinase A) and MMP-9 (gelatinase B) participate in the catalytic cleavage of extracellular gelatine and collagen. In addition, MMP-9 may also exert non-catalytic anti-apoptotic signalling effects [77]. Both MMP-2 and -9 are thought to be directly involved in AAA formation.

Tetracyclines are known to inhibit the activity of MMPs. This action has been attributed to their competitive binding of the catalytic zinc ion needed for MMP activity. Various natural tetracyclines and synthetic analogues have been tested as MMP inhibitors [78,79]. The aim of these studies was inhibition of the breakdown of the extracellular matrix that is thought to promote AAA formation and to facilitate metastasis of tumour cells [80]. The mechanism of action of MMP inhibition has been a rationale to design and produce tetracycline analogues void of antibacterial activity [78,81,82,83]. The fact that the exclusion of this activity might also cause loss of antimitochondrial activity has not been tested or has even been considered [78,83]. Non-antibacterial tetracycline analogues have been tested in a number of human cancer trials [84]. Unfortunately, so far, these studies yielded mostly disappointing results [78,79] and their poor clinical performances as anticancer agents have been stressed [85]. Curci *et al.* examined a series of 15 patients with AAA, of whom 8 patients were treated with an oral dose of 100 mg of doxycycline twice a day for 7 days prior to surgery [86]. Total MMP enzyme activity measurements in post-operative aneurysm tissue failed to show differences.

## 6. Doxycycline in Cell Culture and Animal Models of AAA

Table 2 summarises the main aspects of the studies in which doxycycline has been tested as a possible drug in experimental animal models of AAA. The daily oral or subcutaneous dosage of doxycycline used in the *in vivo* studies varies from 10 to 100 mg/kg. The current dosage for patients in standard doxycycline treatment is approximately 1.5 or 3 mg/kg; however, not the dosage is critical but the serum levels that are reached. These are, unfortunately, lacking in most studies. Only Prall *et al.* showed that their regimes for humans and mice gave comparable blood levels [87].

Doxycycline has been shown to effectively inhibit AAA formation in most reported animal studies (Table 2; [88]). Furthermore, a delay of aneurysm rupture by doxycycline has been demonstrated in a mouse model of Marfan syndrome, a genetic connective tissue disorder [89]. These effects have been ascribed to a reduction of MMP-9 activity with a preservation of elastin in the extracellular matrix [90]. In cultured THP-1 monocytes, stimulated with phorbol ester, decreased expression of MMP-9 mRNA and proprotein have been observed after doxycycline treatment [86]. Even so, in their review of 2008, Baxter *et al.* concluded that additional studies are needed to clarify the role of doxycycline in the progression of aneurysmal disease [8].

**Table 2:** Effect of doxycycline in studies of AAA animal models.

Publication		Doxycycline		No. of Animals	Study Parameters				Effect
1st Author (Year)	Ref.	Dosage	Serum Level		Type	Duration	Methods	Outcomes	
Petrinec, D. (1996)	[90]	12.5 mg bid, s.c.	No data	48 rats	PC	≤14 days	el.-ind. AAA	MMP exp.; MMP act.; expansion	+
Boyle, J.R. (1998)	[91]	1 and 10 µg/mL	NA	8 porcine samples	*In vitro*	13 days	el.-ind. AAA	MMP exp.; MMP act	+
Curci, J.A. (1998)	[92]	n.s., s.c.	No data	52 rats	Open controlled	7 days	el.-ind. AAA	MMP exp.; MMP act.; expansion	+
Prall, A.K. (2002)	[87]	10, 50, 100 mg/kg/d in d.w.	1.4, 2.7, 11.9 µg/mL	n.s. (mice)	Open PC	10 weeks	el.-ind. AAA	MMP exp.; expansion	+
Manning, M.W. (2003)	[93]	30 mg/kg/d in d.w.	No data	60 mice (hyperlipidemic)	PC	5 weeks	ang. II-ind. AAA	Expansion	+
Sho, E. (2004)	[94]	60 mg/kg/d, s.c.; 1.5 mg/kg/d via infusion	No data	n.s. (rats)	PC Controlled	14 days	el.-ind. AAA; periaortic infusion	MMP exp.; expansion; macrophage density	+
Bartoli, M.A. (2006)	[95]	100 mg/kg/d systemic p.o.	0.33 µg/mL	n.s. (mice)	Open comparative	14 days	el.-ind. AAA	Expansion	+
		≤1 mg/kg/d via local infusion	5.6‒7.8 ng/mL	n.s. (mice)	Open comparative	14 days	el.-ind. AAA; localised infusion with osmotic minipump	Expansion	+
Xiong, W. (2008)	[89]	100 mg/kg/d in d.w.	No data	48 mice	Open PC	6 weeks + follow-up	Marfan syndrome	Survival	+
Xie, X. (2012)	[96]	100 mg/kg/d in d.w.	2.3 ± 0.6 μg/mL	25 mice	PC	8 weeks	ang. II-ind. AAA	Expansion	–
Sivaraman, B. (2013)	[97]	2%, 5% and 10% in nanoparticles	NA	n.s. (rat AAA tissue)	*In vitro*	Rats: 2 weeks TC: 3 weeks	el.-ind. AAA	MMP exp.; MMP act	+

Abbreviations: ang. II-ind. AAA, angioptensin II-induced AAA; bid, twice daily; d.w., drinking water; el.-ind. AAA, elastase-induced AAA; MMP exp., MMP gene expression; MMP act, MMP enzyme activity; NA, not applicable; n.s., not specified; PC, placebo-controlled; p.o., oral; s.c., subcutaneous; TC, tissue culture.

Improved efficacy on juxta-aortic delivery of doxycycline has been shown in rats by Sho *et al.* with the aid of a novel prototype infusion system [94]. To avoid dose-related side effects of systemic administration of doxycycline, localised administration with osmotic mini-pumps has been applied in mice by Bartoli *et al.* [95]. In their experiments, the doxycycline level in aortic tissue was extremely low as compared to those in other studies. Despite of the low concentration, the authors claimed that the localised administration suppressed aortic dilatation. The development of doxycycline formulations with nanoparticles may offer another approach for localised, controlled and sustained release in the future [97].

As mentioned above, MMP-9 may prevent apoptosis in a non-catalytic manner [77]. This deserves further attention since apoptosis would have a healing effect on AAA. Induction of apoptosis by tetracyclines has been reported [98]. In this context, an *in vivo* investigation with doxycycline in mice bearing an experimentally induced AAA should be mentioned [87]. In this study, doxycycline serum levels were measured after 5 weeks of treatment. The levels matched those obtained with standard doses in patients (2 to 9 μg/mL of serum) and are within the range of an antimitochondrial effect [13]. Although a decrease in aortic diameter and expression of active MMP-9 and its proprotein were shown following doxycycline treatment, the authors did not investigate an antimitochondrial effect and this can, therefore, not be excluded as the primary cause of the aneurysm growth inhibition.

While in most animal studies doxycycline was administered prior to the initiation of the disease, Xie *et al.* [96] started administration in mice 28 days after induction of AAA. This scenario may be more relevant to patients, where a treatment for established AAA is needed. During the 8 weeks of treatment, expansion rates of AAAs were not reduced, suggesting that doxycycline is not effective in this mouse model of established AAA [96].

## 7. Doxycycline in Clinical Trials of AAA Patients

Table 3 sums up the results of investigations in patients. In most studies, a fixed oral dose of 100 mg twice daily was used. In the oldest study, published in 1999 [99], tissue from five patients treated with doxycycline was compared with that from five untreated patients. MMP-2 and -9 mRNA levels in aneurysmal tissue obtained at the time of surgery were strongly reduced by preoperative treatment as compared to the untreated disease controls. Although the study of 15 patients by Curci *et al.* [86], mentioned in paragraph 5, failed to show differences in MMP-9 enzyme activity, doxycycline-treated patients did show reduced levels of MMP-9 mRNA and protein, as well as a suppressed modification of MMP-2 proprotein in macrophages. Promising results were also obtained in a prospective, double-blind, placebo-controlled pilot study with 32 patients by Morosin *et al.* [100]. The active group received oral doses of 150 mg of doxycycline daily for a period of 3 months. The aneurysm expansion rate in the active group was significantly lower than that in the placebo group. In a similar-sized prospective, double-blind, placebo-controlled, phase-II, multicentre study, no significant change was seen in the aneurysm expansion rate but prolonged administration of doxycycline was associated with a gradual reduction in plasma MMP-9 levels; however, enzyme activities were not measured [101].

**Table 3:** Effect of doxycycline in clinical trials with AAA patients.

Publication		Doxycycline			Study Parameters			
1st Author (Year)	Ref.	Dosage	Serum Level	No. of Patients	Type	Duration	Outcomes	Effect
Thompson, R.W. (1999)	[99]	100 mg 2× dd	No data	10	Open	7 days	Small AAA	+
Curci, J.A. (2000)	[86]	100 mg 2× dd	No data	15	Open controlled	7 days	MMP exp.	+
Mosorin, M. (2001)	[100]	150 mg 1× dd	No data	32	DBPC	3 months +15 months	Expansion, 15 months surveillance	+
Baxter, B.T. (2002)	[101]	100 mg 2× dd	4.62 µg/mL	36	DBPC Phase II	6 months	MMP exp.; expansion	+; −
Prall, A.K. (2002)	[87]	100 mg 2× dd	1.8–9.2 µg/mL	n.s.	Open	6 months	Serum levels in range of those of mice in comparative study (see Table 2)	+
Brown, D.L. (2004)	[102]	20 mg 2× dd	No data	50	DBPC	6 months	Acute coronary syndromes, MMP exp.	No AAA
Hackmann, A.E. (2008)	[68]	100 mg 2× dd	No data	44	DBPC	6 months	MMP exp.; expansion	+; +
Lindeman, J.H.N. (2009)	[20]	50, 100, 300 mg 1× dd	No data	60	SB controlled	2 weeks	MMP exp.; MMP act; leukocyte content	+; ±; +
Meijer, C.A. (2013)	[103]	100 mg 1× dd	No data	286	DBPC	18 months	Expansion	−
Kurosawa, K. (2013)	[10,104]	100 mg 2× dd	No data	248	DBPC	2–3 years (planned)	Study in progress	unkown

Abbreviations: 1× dd, once daily; 2× dd, twice daily; DBPC, double-blind placebo-controlled; MMP act., MMP enzyme activity; MMP exp., MMP gene expression; n.s., not specified; SB, single (investigator) blinded.

Although the study does not concern AAA but acute coronary syndromes, it is interesting to mention the paper by Brown *et al.*, who performed a prospective, double-blind, placebo-controlled pilot study of 50 patients, in which the active group was treated with an oral dose of 20 mg of doxycycline twice daily for a period of 6 months [102]. This relatively low dose, described as “subantimicrobial dose” [105], was chosen to exclude an effect on *Chlamydia pneumoniae* that otherwise might have interfered with the primary experimental goal. Pro-MMP-9 activity, but not MMP-2 activity, was decreased after 6 months [102]. Unfortunately, no data were included on the course and level of doxycycline in serum. For *in vivo* experiments it is important to check if the dose used is not only subantimicrobial but also “subantimitochondrial”. In view of the half-life of doxycycline in humans, a cumulative effect remains conceivable [106]. It might well be that 20 or 50 mg twice daily gives a result comparable with 100 mg twice daily; the latter dose is acceptable however, based on experience with the treatment of bacterial infections and in some of the studies cited here. A serum level between 2 and 5 µg/mL, the range of the antimitochondrial action [13], can certainly be reached at a dose of 20 mg twice daily, assuming a half-life of 18 h [107].

Hackmann *et al.* [68] carried out a double-blind, placebo-controlled trial of 44 patients after endoluminal aneurysm repair. After 6 months of treatment with placebo or 100 mg of doxycycline twice daily, plasma MMP-9 levels were decreased significantly below baseline in the doxycycline group, while there was a non-significant increase in the placebo group. Moreover, based on the endograft used, doxycycline treatment resulted in greater reductions in maximum aortic diameter than placebo treatment.

Recently a trial assessing the efficacy of 100 mg doxycycline daily in 286 patients with small AAAs was reported [103]. This study included approximately 8 times the number of patients involved in previous small positive trials (Table 3). The investigators reported no benefit of doxycycline administered at this dose over 18 months; in fact, AAA growth was significantly greater in patients randomised to the active agent [103]. The dose of doxycycline used in this recent trial was less than in a number of previous investigations [86,99,100,101]. The dose of doxycycline chosen was based on a previous investigation in which the drug was assessed in 60 patients with large AAAs awaiting open aortic surgery [20]. That study suggested that 100 mg of doxycycline daily markedly reduced inflammation within the AAAs of patients randomised to the medication. The drug administration period was very short however at 2 weeks. It is conceivable that a single daily oral dose of 100 mg of doxycycline is not sufficient to maintain adequate doxycycline serum levels for a prolonged period [108]. If the doxycycline serum levels drop too low during the day, then the suggested benefits of the drug outlined in this article may not be able to be realised. A large on-going trial is investigating the benefit of a larger daily dose of doxycycline in patients with small AAAs [10,104]. Overall however there is currently no convincing clinical data that doxycycline benefits patients with small AAAs.

## 8. Direct and Indirect Effects of Doxycycline

The question was raised recently if any of the pharmacological agents tested do really slow the progression of AAA [61]. Lu *et al.* conclude that there remain many undefined complexities regarding the mechanisms and their possible translational consequences in spite of an improved understanding [9]. As discussed in this review, several studies support doxycycline treatment of patients with pre-operative AAA or at high risk of mortality at surgical intervention; however, we strongly question the assumption that clinical improvement with doxycycline can be ascribed to direct inhibition of the expression of MMPs.

Although the ability of doxycycline to chelate zinc may explain the effect on MMP enzyme activity and, possibly, MMP protein levels through destabilisation of its structure, there is no explanation how doxycycline can have an effect on the MMP mRNA levels. Therefore, we postulate that the primary effect of doxycycline is based on its inhibition of mitochondrial protein synthesis, preventing clonal expansion of T-cells and possibly also other proliferating cells in AAA tissue, thus, indirectly, resulting in a decrease of proinflammatory cytokines, MMP-2 and MMP-9 that otherwise damage the vascular stroma [9,28,41]. Whilst there is no direct experimental evidence available that the observed effects of doxycycline on AAA are based on its inhibition of mitochondrial protein synthesis, the finding of Lu *et al.* [11] that aneurysmal lesions contain clonally expanded T cells combined with our own observations in cancer research that doxycycline results in a proliferation arrests of T cells through its inhibition of mitochondrial protein synthesis [72,73] lends strong support to our hypothesis. The resulting changes in cell type composition of the AAA tissue render quantification of mRNA and primary translation products unreliable. Hence, the measured effects of doxycycline on MMP expression are indirect because the antibiotic does not influence nuclear-cytosolic protein synthesis.

We hope that this critical review and our new hypothesis on the role of doxycycline as drug of choice in AAA will spur further research on the possible role of mitochondrial protein inhibition in the treatment of AAA. Although the recent, large, double-blind, placebo-controlled Dutch clinical trial found that a single daily dose of 100 mg of doxycycline did not reduce aneurysm growth [103], we believe that further investigations are justified. A vital demand for success is a permanent serum level of doxycycline at a concentration that effectively inhibits mitochondrial protein synthesis; twice a day a moderate dose may suffice. Such a treatment scenario (100 mg twice daily), or even a higher dosage (200 mg twice daily), is regularly applied to patients with Lyme disease [109,110,111]. Measurement of peak wall stress may be a useful parameter to judge a significant contribution of doxycycline to a better prognosis for AAA [3]. A large on-going clinical trial with a doxycycline dose of 100 mg twice daily, estimated to be completed June 2017, has been announced [10]. Surveillance of serum levels is, unfortunately, not included as secondary outcome [104].
